# The Relationship Between Epistaxis and Stages of Hypertension: A Prospective Observational Study of 250 Cases in Emergency Medicine

**DOI:** 10.7759/cureus.86722

**Published:** 2025-06-25

**Authors:** Shivam Aggarwal, Dikshit Shivgotra, Anil Kumar, Amrit Podder, Jayballabh Kumar

**Affiliations:** 1 Department of Ear, Nose, and Throat (ENT) Head and Neck Surgery, Venkateshwara Institute of Medical Sciences, Gajraula, IND; 2 Department of Physiology, Teerthanker Mahaveer Medical College and Research Center, Moradabad, IND

**Keywords:** anterior epistaxis, blood pressure, hypertension, microvasculature, posterior epistaxis

## Abstract

Introduction: Epistaxis is one of the most common nasal health emergency presentations and is frequently associated with systemic hypertension. As the exact relationship between various stages of hypertension and epistaxis severity and frequency remains poorly defined, in this study we aimed to evaluate the correlation between stages of hypertension and the incidence, type, and severity of epistaxis among patients presenting to the emergency department.

Methods: A prospective observational study was carried out over 12 months in the casualty department of a tertiary care hospital in India in which a total of 250 patients with epistaxis were evaluated for their status of blood pressure and history of hypertension. Blood pressure was classified per the American Heart Association (AHA) guidelines into normal, elevated, stage 1, and stage 2 hypertension. Variables including the type of epistaxis (anterior vs posterior), recurrence, severity, and required interventions were recorded and statistically analyzed using SPSS software version 27 (IBM Corp., Armonk, NY, USA).

Results: Among 250 patients, 48% (120 patients) had stage 2 hypertension, 30% (75 patients) had stage 1, 15% (38 patients) had elevated blood pressure, and 7% (17 patients) had normal readings. Posterior epistaxis was significantly more common in stage 2 hypertensive patients (p < 0.001). Recurrent and severe episodes were also more frequent in stage 2. Multivariate analysis indicated stage 2 hypertension as an independent predictor for posterior and recurrent epistaxis (OR = 3.4, 95% CI = 2.1-5.2).

Conclusion: There is a significant correlation that exists between the severity of hypertension and the type and recurrence of epistaxis. Patients with stage 2 hypertension are at higher risk of developing recurrent nasal bleeding episodes and may need aggressive management.

## Introduction

Epistaxis is one of the most common nasal health emergency presentations and is frequently associated with systemic hypertension. It affects a significant population at least once in their lifetime and accounts for about one in 200 emergency department (ED) visits. Although most cases are self-limiting, a significant proportion require medical intervention [1]. Although the anterior epistaxis is typically benign, the posterior epistaxis often necessitates hospitalization [2]. Hypertension has long been suspected as a contributing factor to epistaxis [3]. The relationship between high blood pressure and epistaxis remains controversial in such a way that several studies have shown a positive correlation between these two, whereas others have failed to demonstrate causality [4,5]. This is why we aimed to address this knowledge gap by prospectively analyzing 250 cases of epistaxis associated with varying stages of hypertension. This study evaluates whether increasing severity of hypertension correlates with more frequent, severe, or posterior epistaxis.

## Materials and methods

Study design and settings 

This prospective observational study was conducted in the casualty department of Teertahnker Mahaveer Medical College and Research Centre, a tertiary care center in Moradabad, Uttar Pradesh, India, over a 12-month period from January 2024 to December 2024.

Inclusion and exclusion criteria

All patients above the age of 18 years, presenting with active epistaxis to the Casualty Department, were documented for their blood pressure status. Patients with any systemic bleeding disorders or on anticoagulant therapy, patients with deviated nasal septum, trauma, nasal tumors or associated with any other risk factors of epistaxis other than hypertension were excluded from the current study. 

Classification of the blood pressure

Blood pressure was categorized based on the American Heart Association (AHA) 2017 guidelines [6]. Each patient's blood pressure (BP) was measured in the emergency department using a calibrated digital sphygmomanometer in a seated position, after a rest period of five minutes.

Collection of data 

A standardized data collection form was used to record the demographics (age, gender), type of epistaxis (anterior vs posterior), duration and frequency of bleeding, previous history of hypertension, BP readings at presentation, comorbidities, type of intervention required (nasal packing, cauterization, surgical ligation), admission and outcomes of the participants. All enrolled participants were signed for a voluntary informed consent proforma for our current study, for which we also obtained Institutional Ethical Clearance Certificate from the Institutional Ethics Committee (IEC) of the Teerthanker Mahaveer University (Ref No: TMU/IEC/2024-25/FACULTY/01B Dated 01-01-2024).

Statistical analysis 

The obtained data were stored in a Microsoft Excel sheet (Microsoft, Redmond, WA, USA) and were analyzed using SPSS software version 27 (IBM Corp., Armonk, NY, USA). Continuous variables were expressed as means ± standard deviations. Categorical variables were expressed as frequencies and percentages. Chi-square tests were used for categorical comparisons. A multivariate logistic regression was performed to identify independent predictors of posterior or recurrent epistaxis. A p-value of <0.05 was considered statistically significant.

## Results

Demographic profile 

Among the 250 patients, 153 (61.2%) were male and 97 (38.8%) were female. The mean age of the participants was 57.4 ± 13.8 years and the most affected age group was 51 to 70 years. Recurrent bleeding was noted in 46 patients (18.4%), with 70% of these in stage 2 hypertensive patients. Severe bleeding (defined as >10 minutes, drop in hemoglobin, or requiring hospitalization) was seen in 39 patients (15.6%), predominantly among those with stage 2 hypertension (28 patients). Out of the 250 patients, nasal packing was carried out for 162 patients (64.8%), cauterization was carried out for 43 patients (17.2%), surgical or arterial ligation was carried out for 12 patients (4.8%) and the remaining 33 patients (13.2%) needed no intervention. Table 1 shows the distribution of hypertensive stages among the study participants, Table 2 clearly shows the type and severity of epistaxis, and Table 3 visualizes the association between hypertension and epistaxis type. We carried out a multivariate logistic regression after adjusting for age, sex, and comorbidities, for stage 2 hypertension (Table 4). The OR for posterior epistaxis was found to be 3.4 (95% CI: 2.1-5.2), p < 0.001 and the OR for recurrence was found to be 2.9 (95% CI: 1.7-4.8), p < 0.001. Table 4 is predicting the likelihood of having stage 2 hypertension, based on two main predictor variables in which outcome (dependent variable) is presence of stage 2 hypertension (yes/no), predictor (independent) variables are posterior epistaxis: Individuals presenting with posterior epistaxis were 3.4 times more likely to have stage 2 hypertension compared to those without posterior epistaxis, after adjusting for age, sex, and comorbidities and recurrent epistaxis: Individuals with recurrent epistaxis were 2.9 times more likely to have stage 2 hypertension. Both associations are statistically significant (p < 0.001), indicating a strong and independent relationship with stage 2 hypertension. It suggests that posterior and recurrent nosebleeds may be strong clinical indicators of underlying severe hypertension, and such presentations in the emergency department should prompt a thorough cardiovascular assessment.

**Table 1:** Distribution of hypertension stages No specific statistical tests are required for descriptive variables. SBP, systolic blood pressure; DBP, diastolic blood pressure.

Stage	Number of Patients	Percentage
Normal (SBP <120 mmHg and DBP <80 mmHg)	17	6.8%
Elevated (SBP 120-129 mmHg and DBP <80 mmHg)	38	15.2%
Stage 1 (SBP 130-139 mmHg or DBP 80-89 mmHg)	75	30.0%
Stage 2 (SBP 140-159 mmHg and DBP 90-99 mmHg)	120	48.0%

**Table 2:** Type and severity of epistaxis Posterior epistaxis was more prevalent among stage 2 hypertensive patients (62.2%). No specific statistical tests are required for descriptive variables.

Type of epistaxis	Number of patients	Percentage
Anterior	160	64%
Posterior epistaxis	90	36%

**Table 3:** Association between hypertension and epistaxis type Analysis were done using Chi-square test (p = 0.0000031 which is < 0.001), Chi-square statistic (χ²) = 28.30, Degrees of freedom (df) = 3

Hypertension Stage	Anterior Epistaxis	Posterior Epistaxis
Normal	15	2
Elevated	31	7
Stage 1	57	18
Stage 2	57	63

**Table 4:** Predictors of stage 2 hypertension: multivariate logistic regression analysis Multivariate logistic regression analysis showing independent associations of posterior and recurrent epistaxis with stage 2 hypertension. Odds ratios are adjusted for age, sex, and comorbidities. A p-value <0.05 was considered statistically significant.

Variable	Odds Ratio (OR)	95% Confidence Interval (CI)	p-value
Posterior Epistaxis	3.4	2.1 – 5.2	<0.001
Recurrent Epistaxis	2.9	1.7 – 4.8	<0.001

## Discussion

This prospective study demonstrates a strong and statistically significant association between the severity of hypertension and the type, recurrence, and severity of epistaxis. The results of this study clearly indicate that stage 2 hypertensive patients were more likely to present with posterior, recurrent, and severe episodes. The posterior epistaxis noted in stage 2 hypertensive patients aligns with anatomical and hemodynamic considerations. Elevated arterial blood pressures may compromise the integrity of posterior nasal vasculature, especially branches of the sphenopalatine artery, leading to more difficult-to-control hemorrhages [7]. Contrary to earlier studies that questioned the causal link between hypertension and epistaxis, our study supports a direct correlation, particularly with increasing stages of hypertension. The prospective design, use of AHA staging, and standardized data collection strengthen the validity of our findings [6,7].

Mechanistic pathophysiological pathways involved in the observed results

Multiple mechanisms might potentiate their involvement in the generation of nasal bleeds. The following might be two possible pathophysiologic mechanisms for the observed results in our study.

Increased Stiffening of the Microvasculature

Various studies have already evidenced that increased blood pressure is correlated with increased vascular stiffening. The more stiffening, the greater the alteration in the architecture of the vessels. The altered architecture of the microvasculature due to high blood pressure may be the underlying mechanism for the development of epistaxis [8,9].

Altered Vascular Proteins

Alongside stiffened arteries, hypertension is also associated with altered vascular proteins such as angiotensinogen, vascular endothelial growth factor (VEGF), etc. The altered level of these proteins for a longer duration may be the fundamental molecular mechanism involved in the process of development of nasal bleeds associated with high blood pressure [10,11].

Role of lifestyle changes in the prevention of epistaxis

Studies have already shown that lifestyle modifications such as pranayama and other breathing exercises play significant roles in keeping the blood pressure within a normal limit. As high blood pressure directly correlates with development of epistaxis, a regular breathing exercise practice might be helpful in the prevention of nosebleed [12].

Limitations of the present study

The current study is a single-center study, which potentially limits generalizability; the BP readings were taken at the time of the clinical presentation of nosebleed which may also reflect stress-induced elevations and potential unmeasured confounders (e.g., undiagnosed coagulopathies, medication noncompliance) cannot be ruled out in the participants. We consider these as some of the limitations of our study.

## Conclusions

The findings from the current study suggest a clear and progressive relationship between stages of hypertension and the risk, type, and severity of epistaxis. Emergency physicians may consider stage 2 hypertension as a red flag in patients presenting with posterior or recurrent nosebleeds. Better control of blood pressure may serve as a preventive measure for recurrent epistaxis. Hence, we recommend routine BP monitoring and aggressive management in patients with epistaxis. Further multi-centric and longitudinal studies are also recommended to explore causality of the patients presenting with epistaxis.
